# Map2k5-Deficient Mice Manifest Phenotypes and Pathological Changes of Dopamine Deficiency in the Central Nervous System

**DOI:** 10.3389/fnagi.2021.651638

**Published:** 2021-06-08

**Authors:** Yumeng Huang, Pei Wang, Rodrigo Morales, Qi Luo, Jianfang Ma

**Affiliations:** ^1^Department of Neurology, Institute of Neurology, Shanghai Jiao Tong University Medical School Affiliated Ruijin Hospital, Shanghai, China; ^2^Department of Neurology, McGovern Medical School, The University of Texas Health Science Center at Houston, Houston, TX, United States; ^3^Centro Integrativo de Biologia y Quimica Aplicada (CIBQA), Universidad Bernardo O’Higgins, Santiago, Chile

**Keywords:** MAP2K5, mouse model, dopamine, sensorimotor phenotypes, restless legs syndrome, Parkinson’s disease

## Abstract

MAP2K5, a member of the MAPK family, is associated with central nervous system disorders. However, neural functional of Map2k5 from animal models were not well examined so far. Here, we established a Map2k5-targeted knockout mouse model to investigate the behavior phenotypes and its underlying molecular mechanism. Our results showed that female Map2k5 mutant mice manifested decreased circadian-dependent ambulatory locomotion, coordination, and fatigue. Male Map2k5 mutant mice displayed impairment in open field exploration and prepulse inhibition of acoustic startle response (ASR) when compared with wild-type controls. Furthermore, Map2k5 mutant mice showed a decreased dopaminergic cell survival and tyrosine hydroxylase levels in nigrostriatal pathway, indicating a crucial role of MAP2K5 in regulating dopamine system in the central nervous system. In conclusion, this is the first study demonstrating that Map2k5 mutant mice displayed phenotypes by disturbing the dopamine system in the central nervous system, implicating Map2k5 mutant mouse as a promising model for many dopamine related disorders.

## Introduction

MAP2K5 (mitogen-activated protein kinase kinase 5, also known as MEK5) has been associated with various diseases including cardiovascular diseases, cancers, and central nervous system disorders (Appari et al., 2017; Liu et al., 2017; Castro et al., 2019). In the central nervous system, some polymorphisms of MAP2K5 have been associated with restless legs syndrome/William–Ekbom disease (RLS/WED) and neuropsychologic disorders (Winkelmann et al., 2007; Yang et al., 2011; Li et al., 2017). In addition, studies also suggested its etiologic or therapeutic role in amyotrophic lateral sclerosis, Parkinson’s disease (PD), schizophrenia, suicidal behavior, and others (Gan-Or et al., 2015; Jo et al., 2017; Kang et al., 2018; Cabrera-Mendoza et al., 2020). Although the role of Map2k5-Erk5 in cardiovascular diseases and cancer has been mostly clarified, the molecular mechanism of Map2k5 in the central nervous system remained largely unknown.

MAP2K5 is a member of the MAP kinase family, and it is involved in growth factor-induced cell proliferation, survival, and differentiation (Dinev et al., 2001; Liu et al., 2006). It specifically activates ERK5 by phosphorylation on threonine and tyrosine residues (Johnson and Lapadat, 2002). The MAP2K5–ERK5 pathway regulates many transcription factors including MEF2, c-MYC, and NF-κB (English et al., 1998; Kato et al., 2000; Simões et al., 2015), which were identified as essential in cells’ survival and development. Moreover, the role of the MAP2K5–ERK5 pathway in neurons’ proliferation, differentiation, and apoptosis were demonstrated in many studies, and included activity in cortical, dopaminergic, and GABAergic neurons (Liu et al., 2006; Zou et al., 2012; Parmar et al., 2015; Ding et al., 2020). Target deletion of MAP2K5 in animals may help us to discover its role in biochemical processes and gross functions. However, there are few studies using animal models focused on the role of MAP2K5 in the central nervous system. This is probably due to the fact that fully knockout Map2k5 mice died at early embryonic periods from heart deficits (Wang et al., 2005).

Based on the finding that polymorphisms (rs12593813, rs11635424, and rs868036) in MAP2K5 were associated with a reduced risk of RLS/WED, a neurological disorder characterized by dopamine and iron dysfunction, we established a targeted deletion of Map2k5 in mice. Specifically, we focused on the deletion of exons 18–22, which harbor three risky SNPs. This model was hypothesized to be useful in exploring the role of this protein in the central nervous system *in vivo*. We found that the homozygous Map2k5-deficient mouse died at mid gestation, similarly, as previously reported (Wang et al., 2005). Heterozygous female Map2k5-deficient mouse manifested decreased locomotion, impaired coordination, and was prone to fatigue. Interestingly, these phenotypes are contrary to RLS/WED and, in some aspects, similar to PD (another movement disorder involving dopamine system disturbance). Furthermore, we found reduced dopaminergic neurons and decreased tyrosine hydroxylase (TH) levels in female Map2k5-deficient mice compared with wild-type (WT) mice in nigrostriatal dopaminergic pathway, which may explain the motor dysfunction in female Map2k5-deficient mice. To our knowledge, this is the first *in vivo* study that demonstrated the relationship of dopaminergic system disturbance with the Map2k5–Erk5 pathway.

## Materials and Methods

### Map2k5^em41^Cd71454 Mice

**FIGURE 1:**
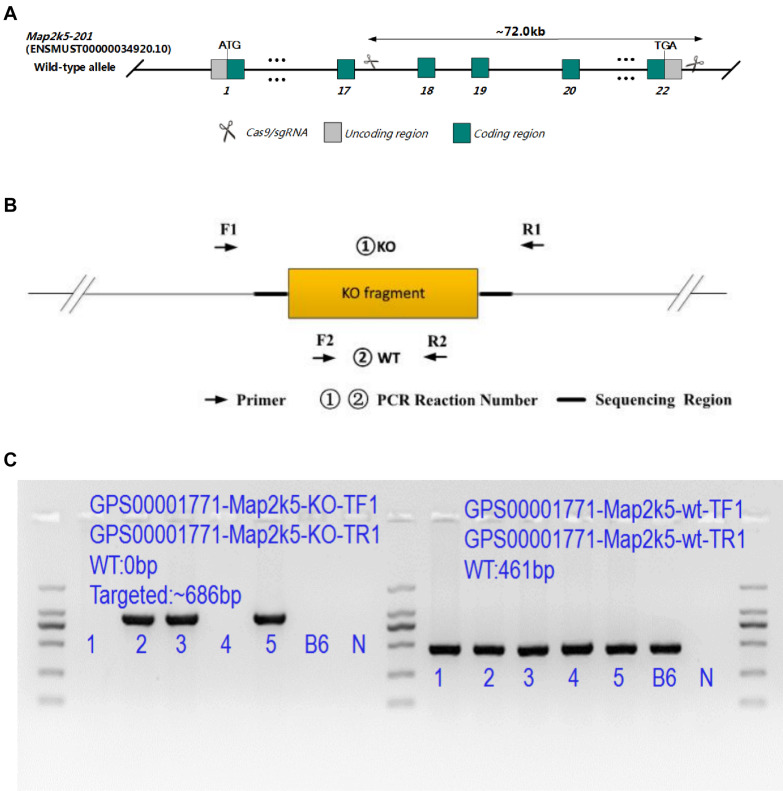
**(A)** Targeting strategy of Map2k5-targeted allele. Green boxes represent coding regions, gray boxes represent noncoding exon regions, and scissors represent the targeted area of Map2k5 between exons 18 and 22. **(B)** Primers designed for genotype identification of Map2k5^+/–^ heterozygous and wild-type (WT) mice. **(C)** PCR results of some Map2k5^em41^Cd71454 mice.

Animal experiments were performed in accordance with the guidelines and policies of the Association for Assessment and Accreditation of Laboratory Animal Care International (AAALAC International), China National Accreditation Service for Conformity Assessment (CNAS), and Laboratory Animal Resource Center of Shanghai Jiao Tong University School of Medicine. Map2k5^em41^Cd71454 mice were created in Nanjing, China (GemPharmmatech Co., Ltd.) via CRISPR/Cas9 technique on a C57B6/JGpt (GemPharmmatech Co., Ltd.) background. In this strain, based on the findings that most reported SNPs in MAP2k5 of RLS/WED [rs12593813 (A/G), rs11635424 (A/G), and rs868036 (T/A)] were located in the intron area of gene Map2k5 between exons 17–18 and exons 19–20 (RefSeq: NM_011840), transgenic mice with Map2k5 exons 18–22 deletion were constructed. Two sgRNAs on both sides of the targeted region were designed specifically (Figure 1A) (sgRNA1: GGCTCCGTAAGTGTCCACATGGG; sgRNA3: TGCAGGCACGGGCCTTTGACTGG) and constructed by using MEGAshortscript^TM^ Kit (Thermo Fisher Scientific, Waltham, MA, United States). In order to microinject Cas9 protein and sgRNA into fertilized eggs, male C57BL6/J mice were individually caged for 1 week before mating. Female C57BL6/J mice were given 5 IU of serum gonadotropin and 5 IU of human chorionic gonadotropin (HCG) to pregnant horses for superovulation. After C57BL6/J male and female mice were injected with HCG, the fertilized eggs of male and female mice were taken for mating. SgRNA (40 ng/μl) and Cas9 protein (40 ng/μl) were injected into the cytoplasm of the fertilized egg at the single-cell stage using continuous flow injection microinjection. The surviving two-cell embryos were implanted into the fallopian tube of a pseudo-pregnant female. The generated mice (F0) were analyzed in the born pups. F0 mice were mated with C57BL6/J wild-type mice to gain the obtained F1 mice. Their Map2k5 with exons 18–22 deletion sequencing were examined by Sanger Sequencing (Supplementary Material 1). To exclude the off-target effect, genetic sequencing of high frequent loci of each sgRNA (sgRNA1 and sgRNA3) were predicted by CRISPOR (Concordet and Haeussler, 2018)^1^ and confirmed by Sanger sequencing (Supplementary Material 2).

All offspring were identified by genotyping tail DNA (Accili et al., 1996). KO (knockout) primers (5′ GATGAA GCAAAGGGTGACGATAG 3′ and 5′ CTTGGGAAGGCAAGC ACTACTC 3′, 940 bp) and WT primers (5′ ACATTCCA TCCTACCTCAGCCTAG 3′ and 5′ CCTACTTGCTTGGCTTCA GTCTG 3′, 461 bp) were used for this purpose (Figure 1B). F1 mice were interbred to obtain F2 homozygous, while homozygous mutants were examined dead prenatally after. Large quantities of F3 experiment’s mice were interbred with F2 heterozygous mice (some PCR genetic identification results are shown in Figure 1C). In all experiments, heterozygous Map2k5^+/–^ mice and their WT littermates were used, as homozygotes were embryonic lethal.

### mRNA Prepared and Semiquantitative Reverse Transcriptase -PCR

Total RNA was extracted from homogenized frozen brain, heart, and spleen tissue followed by TRIzol^TM^ Reagent (Invitrogen, Carlsbad, CA, United States) extraction protocols. Reverse transcription was performed using PrimeScript^TM^ RT reagent Kit with gDNA Eraser [TAKARA Biomedical Technology (Beijing) Co., Ltd., China] and qRT-PCR using TB Green^®^ Premix Ex Taq^TM^ II [TAKARA Biomedical Technology (Beijing) Co., Ltd., China] with the SYBR Green Method (Applied Biosystems, Foster City, CA, United States). Oligonucleotides_Map2k5 (5′ AAG CGA TGA AGA GAT GAA G 3′ and 5′ ATG TAT GTT CCG TTC CCC A 3′) targeted the dominant Map2k5 mRNA. The primers of the glyceraldehyde-3-phosphate dehydrogenase (Gapdh) gene (5′ AAA TGG TGA AGG TCG GTG TG 3′ and 5′ AGG TCA ATG AAG GGG TCG TT 3′) were used as the reference gene—glyceraldehyde-3-phosphate dehydrogenase (Gapdh). Run method: (a) 30 s at 95°C; (b): 40 cycles of 5 s at 95°C, 34 s at 64°C; (c)15 s at 95°C at 1.6°C/s, 1 min at 65°C at 20°C/s, 15 s at 95°C at 0.05°C/s.

### Behavioral Studies

Map2k5^+/–^ mutant mice and WT littermates were grouped by genotype and gender. Behavior studies were carried out from 9 to 25 weeks, and their body weights were tracked by an electronic weigher (NV621ZH, OHAUS, Parsippany NJ, United States) from 6 to 27 weeks.

#### Rotarod

Rotarod (ZH-600B, Anhui Zhenghua Co., Huaibei, China) was used to measure the motor coordination, learning ability, and fatigability of the mice. A rotarod device was placed in an SPF experiment room. Each mouse was placed in the room for 30 min for adaptation before running the experiments. Both WT and Map2k5^+/–^ mutant mice aged 9 weeks were tested in the rotarod. During the learning period, both groups of mice were trained three times per day for continuous 3 days at a speed of 1 rpm for 30 s and then at an accelerating speed from 1 to 10 rpm by 1 rpm/30 s for 300 s. On the day 4, all mice were tested on the rotarod at an accelerating speed of from 4 to 30 rpm for 500 s and 30-min resting interval between each trial. The latency to fall off the rotarod was recorded.

#### Open Field

Open field device (XR-XZ301, Shanghai Xinruan Co., Shanghai, China) was placed in an SPF experiment room. It had infrared sensors, and the center was exposed to bright illumination (90–120 lx). All experimental mice were aged 12 weeks. Each mouse was placed in the middle of one edge of the open field box and recorded for 20 min. The activity of each mouse from 0 to 5 min was used for analysis. TopScan Lite Analysis Software (TopScan Lite, CleverSys) was used to collect and analyze data.

#### Limbs’ Strength

Fore and four limbs strength were tested by the ability to hold on to a grip sensor (Bioseb G3, Chaville). Mice aged 13 weeks were tested. Each mouse was placed on a wire screen with their fore limbs or four limbs grasping to the grip. The grip sensor recorded the force of a metal grate being pulled in grams. We conducted three trials per mouse. The average strength of three trials was analyzed.

#### Acoustic Startle Response (ASR) and Prepulse Inhibition (PPI)

ASR and PPI apparatus Startle Response System (SR-LAB, San Diego, CA, United States) were used. We followed the standard protocols published by the International Mouse Phenotyping Consortium (IMPC) on the website^2^. Mice aged 17 weeks were tested. Each experiment was carried out at the late active phase from 7:00 to 8:00 a.m. The session was initiated with a 5-min acclimation period (only background noise was used). In addition, it had an option to acclimate to the startle pulse in which 110–120 dB/40–60 ms of white noise was presented alone, five times. These were excluded from the statistical analysis. Different prepulse trials of 20-ms duration of white noise stimuli were presented alone (PP1 = 72 dB, PP2 = 78 dB, PP3 = 84 dB, or PP4 = 90 dB) and preceded the pulse by 70 ms (PP1 + pulse, PP2 + pulse, PP3 + pulse, or PP4 + pulse) to derive the pre-pulse inhibition response. Startle pulse trials were started where 120 dB/50 ms of white noise was presented alone. No stimulus trials in which only background noise was presented were used to measure baseline movement of the animal in the chamber. Background noise was 65–70 dB. Startle responses were recorded and analyzed.

#### Homecage Locomotor Activity

Individual mice aged 25 weeks were placed in the Laboratory Animal Monitoring System (CLAMS, Columbus Instruments) for 24-h adapting followed by 48-h test. Data were collected and analyzed every 20 min in the system and resulted in 63 readings per individual a day. Total activity (TOT) and ambulatory activity (AMB) were measured based on the infrared beam breaks.

#### Immunofluorescence Staining

The cohorts included three female mice of each group aged postnatal 18 days (P18d), 21 weeks, 28 weeks, and 38 weeks. Mice were perfused with 4% paraformaldehyde (PFA) in phosphate-buffered saline (PBS) and sacrificed. Mouse brains were isolated on ice and fixed in 4% PFA for at least 24 h. Brain tissues were embedded with paraffin and serially cut into 5-μm-thick coronal sections. Brain sections of SNc and striatum were dewaxed by dimethylbenzene and gradient ethanol solutions. Citrate antigen retrieval solution was used, heated for 6 min at 65°C, with 1-min interval, and reheated for 6 min at 65°C for antigen retrieval. Every four continuous sections of SNc and striatum regions were stained in each mouse. The sections of mouse brains were immunostained at 4°C overnight with primary anti-tyrosine hydroxylase antibody (1:500, MAB318, Millipore Co., Massachusetts, United States), MAP2K5 (Concordet and Haeussler, 2018). Antibody (1:200, sc-135986, Santa Cruz Biotechnology, Inc., Northern California, United States), followed by Alexa Fluor 594-conjugated secondary antibodies and 488-conjugated secondary antibodies (1:200, YEASEN Biotech Co., Ltd., Shanghai, China). Each section was incubated by 4′,6-diamidino-2-phenylindole dihydrochloride (1:1,000, DAPI, Sigma-Aldrich, St. Louis, United States) for 20 min and treated with Antifade Mounting Medium (Beyotime Biotechnology, Shanghai, China). Images of each section were captured via fluorescence microscopy (Nikon Eclipse C1, Nikon, Tokyo, Japan). For each brain, three sections of substantia nigra pars compacta (SNc) and striatum were analyzed. The TH^+^ stained cell counts and mean immunofluorescence intensity per section were measured using ImageJ (NIH).

#### Western Blot

Map2k5^+/–^ mutant mice and WT littermates (female, aged 21 weeks, *n* = 4 per group) were perfused with PBS and sacrificed. Brains were then dissected quickly within 10 min and snap frozen in liquid nitrogen. Proteins were prepared from the ventral midbrain (Yun et al., 2018) including nigrostriatal dopaminergic neurons. Cytoplasmic proteins were extracted by RIPA Lysis Buffer (Strong) (Beyotime Biotechnology, Shanghai, China) supplemented with Complete Protease Inhibitor Cocktail (10×) and PhosSTOP^TM^ Cocktail (10×) (Roche Ltd., Basel, Switzerland). Western blots were performed using sodium dodecyl sulfate polyacrylamide gel electrophoresis and nitrocellulose membranes. Primary antibodies included anti-tyrosine hydroxylase Ab (1:1,000, MAB318, Millipore Co., Massachusetts, United States), Erk5 Antibody (1:1,000, #3372, CST Ltd., Boston, United States), Phospho-Erk5 (1:1,000, Thr218/Tyr220), Antibody (1:1,000, #3371, CST), MAP2K5 (Concordet and Haeussler, 2018). Antibody (1:200, sc-135986, Santa Cruz Biotechnology, Inc., Northern California, United States), and GAPDH (D16H11) XP^®^ Rabbit mAb (1:1,000, #5174, CST). Signals were detected with an ECL agent (Millipore). Blotting images were acquired and quantitatively analyzed by Image Lab Software (Bio-Rad, CA, United States).

#### High-Performance Liquid Chromatography (HPLC)

Striatal samples of 21-week-aged female mice were prepared for neurochemical measurements via HPLC analysis (Bionovogene Co., Ltd., Suzhou, China). The tissue was homogenized in 10% methanol formic solution–ddH2O (1:1) (200 μ) and centrifuged at a speed of 12,000 rpm at 4°C for 5 min. One hundred microliters of supernatant and 100 ppb of double isotopes of L-valine (C13) L-phenylalanine (C13) internal standards (100 μl) were mixed and passed through a 0.22-μm filter membrane.

Samples were injected in an ACQUITY UPLC^®^ BEH C18 HPLC column (2.1 × 100 mm, 1.7 μm, Waters Co.) at 40°C. The mobile phase consisted of A-50% methanol with 0.1% formic acid and B-10% methanol with 0.1% formic acid. Compounds were measured with Analyst Software (AB SCIEX). The neurotransmitter metabolite peak areas were integrated and quantified against standard samples. The standard curves *r* > 0.99, and the relative standard deviation (RSD) of the samples was < 9%. Phenylalanine (Phe), tyrosine (Tyr), DOPA, dopamine (DA), epinephrine (Epi), glutamine (Gln), acetylcholine (ACh), serotonin, and 5-HIAA were measured. Results were normalized to the weight of each tissue and reported in μg/g.

### Statistics

Analyses of mouse data were conducted via SPSS 26 (IBM). The test for genotype and gender-independent factor interactions of behavior studies were tested by two-way ANOVA followed by Tukey’s *post hoc* tests for multiple comparisons in our behavior tests. Test for genotype effect of biochemical studies was conducted via Student’s *t*-test, two-tailed analysis. A *p*-value of 0.05 was set as statistically significant.

## Results

### Characterization of Homozygous Map2k5^–/–^ Mutant Embryos That Died at Mid-Gestation

Map2k5^em41^Cd71454 heterozygous Map2k5^+/–^ mice were viable and fertile. By tracking the body weight of Map2k5^+/–^ and WT mice through 6–27 weeks, it was found that different genotypes exert no influence on body weight of mice, except for male mice at 11 weeks (Figure 1 in Supplementary Material 3).

However, Map2k5^em41^Cd71454 homozygous Map2k5^–/–^ mice died at mid-gestation with gross development retardation. At E9.5 (gestation day 9.5), homozygous Map2k5^–/–^ mutant embryos showed normal phenotype. However, 9 out of 56 (16.67%) homozygous Map2k5^–/–^ embryos displayed developmental retardation at E10.5. Moreover, three homozygous Map2k5^–/–^ out 32 embryos at E11.5 (10.71%) were found dead, 0/11 live embryos were found at E12.5, and 0/32 were found at E13.5. We deduced that Map2k5^em41^Cd71454 strain’s homozygous Map2k5^–/–^ mutant embryos with exons 18–22 depletion died at approximately E11.5. This outcome was similar to the one found in a previous study reporting that Map2k5^–/–^ mutant homozygous mice die at E10.5 due to a defect in fetal cardiogenesis (Wang et al., 2005). Thus, Map2k5^+/–^ heterozygous mice and their WT littermates were used in the postnatal experiments.

### Map2k5^+/–^ Heterozygous Mice Expressed Decreased Levels of Map2k5 mRNA in Different Organs

**FIGURE 2:**
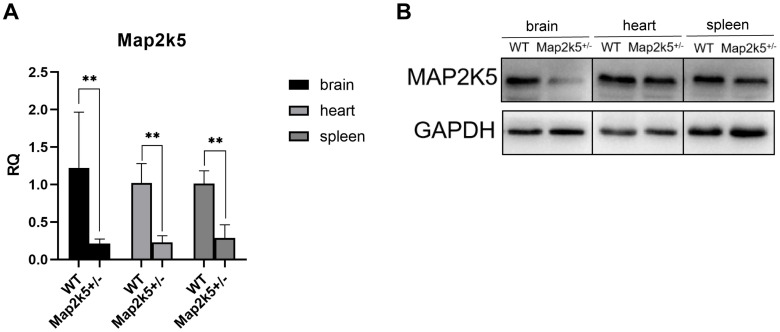
**(A)** Map2k5^+/–^ heterozygous mice expressed 20–30% relative quantitative (RQ) levels of intact Map2k5 mRNA in the brain, heart, and spleen (*t*-test, *n* = 6). Bars represent means with standard errors of the mean (SEM). **(B)** Western blot of Map2k5 expressions in different tissues (brain, heart, and spleen) in WT and Map2k5^+/–^ mice. **P < 0.01.

To assess how effective the Map2k5 exons 18–20 gene deletion was on terminating transcription, the mRNA expression level of Map2k5^+/–^ mutant mice was detected in different organs including the brain, heart, and spleen via qRT-PCR. We found a significant decreased expression levels of Map2k5 mRNA in all three different tissues (brain, heart, and spleen). Specifically, 20–30% relative quantitative levels of Map2k5 mRNA were expressed in Map2k5^+/–^ mutant mice compared with WT mice in the brain, heart, and spleen (referred to GAPDH) (*P* = 0.009, 0.007, 0.007, respectively, Figure 2A). Western blots of corresponding tissues were examined, which displayed a significant decrease in Map2k5 expression in the brain, and a less decrease in the heart and spleen (Figure 2B).

### Map2k5^+/–^ Mice Displayed Sex-Dependent and Circadian-Related Motor Dysfunction

Many movement disorders are circadian related. Based on the fact that RLS/WED is a disease usually presenting clinical signs in the evening and that women have higher risk than men^1^, we explored the profile of motor function in Map2k5^+/–^ mutant mice by performing a series of behavioral tests including open field test, rotarod test, and 12 h–12 h cycle infrared locomotor activity pattern. All these tests were done considering animal sex to assess for potential differences at this level. To exclude the effect of muscle strengths in the motor test results, we examined the force of four and fore limbs in Map2k5^+/–^ and WT mice.

**FIGURE 3:**
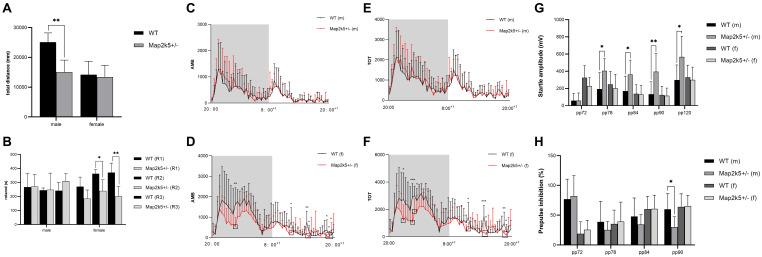
**(A)** Open field test to measure volitional locomotion. Male Map2k5^+/–^ mice displayed a decreased total distance compared with WT mice in traveled in open field test (P = 0.004, *n* = 4). **(B)** Rotarod test. Female Map2k5^+/–^ mice have impaired coordination ability in rounds 2 and 3 (*P*_1_ = 0.157, *P*_2_ = 0.036, *P*_3_ = 0.003, *n* = 4). **(C–F)** Total activity (TOT) and ambulatory activity (AMB) with 12 h (light)–12 h (dark) cycle in both male **(C,E)** and female mice **(D,F)**. AMB and TOT were decreased significantly in female Map2k5^+/–^ mice during their light phase (rest phase) (*n* = 8). **(G,H)** Acoustic startle response (ASR) and prepulse inhibition (PPI) of WT and Map2k5^+/–^ mutant mice. **(G)** ASR increased in male Map2k5^+/–^ mutant mice at 78, 84, 90, and 120 dB (*P* = 0.035, 0.029, 0.006, and 0.019, respectively). **(H)** Prepulse inhibition (PPI) was decreased in male Map2k5^+/–^ mutant mice at 90 dB (*P* = 0.024, *n* = 6). Results are plotted as mean with SEM. Bars represent means with SEM. *P < 0.05, **P < 0.01.

Male Map2k5^+/–^ mice manifested a decreased total distance traveled in the open field test (P = 0.004) when compared with WT mice, while female Map2k5^+/–^ mice displayed no differences compared with the WT controls (Figure 3A). There was also a trend, but not a statistically significant difference, between different genotypes in which male Map2k5^+/–^ mice exhibited decreased central zone duration (*P* = 0.075, figure not shown). These results indicated a different motor response to the new environment of Map2k5 mutant male mice. Previous studies showed that mice exploration behaviors could be influenced by hormones considering the open field cage as a new environment (Lalonde and Strazielle, 2017). Our results implied that male Map2k5+/- mice might display less motivation to explore and locomotion in the open field test.

As a measurement of motor coordination, learning, and fatigability, we evaluated the latency to fall from an accelerating rotarod three times on the test day following a 3-day learning period. It is important to consider that the three times repetition used in this test implies fatigue levels in each mouse. Female Map2k5^+/–^ mutant mice displayed decreased latency to fall at the second and third rounds at the test day, and spared the first round of tests (*P*_1_ = 0.157, *P*_2_ = 0.036, *P*_3_ = 0.003, Figure 3B), suggesting increased fatigue propensity, impaired coordination and learning abilities, while male Map2k5^+/–^ mice displayed no differences compared with WT mice. This indicated that Map2k5 deficiency in female mice may induce fatigue and poor coordination, similar to some motor symptoms presented in PD.

To analyze potential differences of volitional locomotion of Map2k5^+/–^ mutant mice subjected to a circadian variation, we collected motor data per 20 min in a cage equipped with infrared beams (divided a whole day into 63 equal periods). Total activity (TOT, which includes when animals scratches themselves and move their limbs without locomotion) and ambulatory activity (AMB, which only includes their body movement) were recorded with a 12 h (light)–12 h (dark) cycle for a day. Through this method, we found that there was interaction between genders and genotypes (*p* = 0.043); the main effect of genotype on both AMB and TOT in female mice was significant. Specifically, AMB and TOT of female Map2k5^+/–^ mutant mice decreased significantly during their sleeping time (light-on phase, Figures 3D,F), implying a hypokinetic and bradykinetic phenotype (Kim et al., 2016; Langley et al., 2017). However, male Map2k5^+/–^ mutant mice did not display any differences compared with their WT littermates (Figures 3C,E). This provided us with a gender-dependent, circadian-related movement dysfunction profile, contrary to RLS/WED symptoms to some extent (Allen et al., 2014).

When considering the gender-dependent different results of 12 h–12 h locomotor activity trace [significant decreased sleeping body movements (AMB and TOT) only in female mice] and open field tests (only male mice showed a significant reduction in total distance in open field), we thought that as a novel environment exposure, mice were also experienced in the open filed apparatus, the behavior found in male mice indicated stress-based movement in these mice (Lalonde and Strazielle, 2017; Zhang et al., 2019), whereas 12 h–12 h locomotor activity trace data were more similar to the nature pattern of movement, including circadian-distinctive manners. Therefore, male Map2k5-deficient mice motor dysfunction can be interpreted into a stress-based alteration, while female Map2k5 deficient mice movement phenotypes are basal changes due to motor-control pathway dysfunctions.

To discard the effect of limbs’ strength on the behavioral performance, each mouse’s fore and four limbs’ strengths were tested. The results showed that the limbs’ force has no difference between WT and Map2k5^+/–^ mutant mice in both male and female subjects (Figure 2 in Supplementary Material 3).

### Male Map2k5^+/–^ Mutant Mice Display Sensorimotor Gating Deficits

The main complaint of+oldd unpleasant feelings displayed by RLS/WED patients is hard to evaluate in mice. In order to measure this phenotype, we tested the sensorimotor gating levels on each group of mice. The sensory response adaptation was recorded by acoustic startle response (ASR) and prepulse inhibition (PPI) tests. Male Map2k5^+/–^ mutant mice represented a decreased ASR at 78, 84, 90, and 120 dB (*P* = 0.035, 0.029, 0.006, and 0.019, respectively, Figure 3G), and PPI results implied sensorimotor gating deficits at 9 0dB (*p* = 0.024, Figure 3H). This implied that male Map2k5^+/–^ mutant mice displayed deficits on sensory stimulus, but not female mutant mice. ASR and PPI tests examined the intact function of the vestibular system, auditory system, ventrolateral tegmental nucleus, pontine reticular formation, and their interactions (Yeomans et al., 2002). In addition, there is also evidence showing that the amygdala, accumbens nuclei, and other limbic systems contribute to the performance of ASR and PPI (Frau et al., 2019). Considering the results we got from open field and PPI, where only male mutant mice displayed stress-related sensorimotor deficits, and taking in account that both tests involve the accumbens nucleus, amygdala nucleus, and mesolimbic dopamine system (that participate in many psychiatric disorders, such as schizophrenia, depression, obsessive–compulsive disorder, psychological substance-induced behavior, and so on), we hypothesized that sex hormone levels might affect the behaviors of Map2k5^+/–^ mutant mice where male mice was prone to manifest stress-related responses (Pittenger et al., 2016; Khan and Powell, 2018; Liu et al., 2018; Bilel et al., 2019; Tapias-Espinosa et al., 2019).

### The Quantity of SNc and Striatum TH^+^ Cells Were Reduced in Map2k5^+/–^ Mutant Mice

**FIGURE 4:**
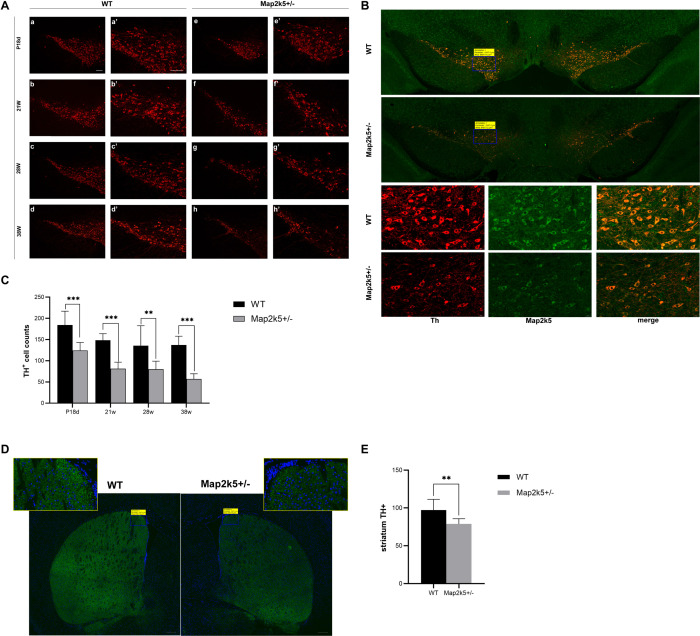
**(A)** Coronal sections of substantia nigra pars compacta (SNc) were prepared from P18d, 21, 28, and 38 weeks and immunostained with an anti-TH antibody. Map2k5^+/–^ mutant mice had decreased TH^+^ cell number in SNc at P18d, 21, 28, and 32 weeks. (a–d) Immunostained TH^+^ cells in SNc of Map2k5^+/+^ WT mice (100x). (e–h) TH^+^ cells in SNc of Map2k5^+/–^ mutant mice (100×). (a’–h’) High-magnification images (200 ×) of the corresponding section in (a–h). Scale bar, 100 μm. **(B)** TH^+^ and Map2k5^+^-stained cells, and merged pictures of Map2k5^+/–^ mutant and WT mice in SNc of at 38 weeks. Low-magnification images (50×) and high-magnification images (400×). 200 μm (50×), 20 μm (400×). **(C)** TH+ cell counts in SNc were reduced in female Map2k5^+/–^ mutant mice compared with WT mice at P18d, 21, 28, and 38 weeks (*P*_1__8d_ < 0.001, *P*_2__1w_ < 0.001, *P*_2__8w_ = 0.005, *P*_3__8w_ < 0.001). **(D)** Coronal sections of striatum immunostained with anti-TH antibody. We found decrease immunofluorescence in Map2k5^+/–^ mutant mice compared with WT mice. Low-magnification images (50 ×) and high-magnification images (630×). Scale bar, 200 μm (50×), 20 μm (630×). **(E)** Mean immunofluorescence intensity of each slice was analyzed. There was decreased TH density in Map2k5^+/–^ mutant mice (*P* = 0.006). Results are plotted as mean with SEM. Bars represent means with SEM. **P < 0.01, ***P < 0.001.

Based on the behavior tests’ results found in female mice, we thought that the phenotype of bradykinesia, poor coordination, and fatigue was similar to the motor dysfunction caused by nigrostriatal pathway deficits. Dopaminergic neurons are crucial for the nigrostriatal pathway, so we assessed the levels of dopaminergic neurons by immunostaining of TH protein (the rate-limiting enzyme in the dopamine synthesis pathway) in SNc. We assessed the TH^+^ cell counts in the SNc region of the mice brain. TH^+^ cells were stained and captured by florescence microscopy (Figure 4A), and a montaged image of Map2k5-stained section was captured, which indicated a synchronous declination of Map2k5 and TH protein (Figure 4B). Results showed a decreased SNc TH^+^ cell number in female Map2k5^+/–^ mutant mice at P18d, 21 weeks, 28 weeks, and 38 weeks compared with WT mice (P_1__8d_ < 0.001, P_2__1w_ < 0.001, P_2__8w_ = 0.005, P_3__8w_ < 0.001, Figure 4C). The reduction of TH^+^ cells in SNc suggested that MAP2K5 might be involved in the survival of dopaminergic cells in SNc early in life. At P18d, the TH^+^ cells of Map2k5^+/–^ mutant mice were 70% of their WT littermates, 50–60% at 21 and 28 weeks, while this number declined to about 40% at age by 38 weeks. Thus, Map2k5^+/–^ deficiency might disturb the dopamine system in SNc, and the effect of Map2k5^+/–^ deficiency on losing dopaminergic neurons increased with aging.

SNc dopaminergic neurons project to the striatum forming the nigrostriatal pathway and facilitate movement via direct and indirect pathways. We conducted immunostaining of TH+ protein in the striatum of 21-week female mice to evaluate the dopaminergic neural fibers’ density in this area, where DA is released and acts on the postsynaptic membrane. Immunofluorescence images of the striatum displayed a reduction in TH intensity (Figure 4D). Mean immunofluorescence intensity was calculated for each tissue slice, showing a significant decrease in dopaminergic neuron fibers’ density in female Map2k5^+/–^ mutant mice compared with WT mice (*p* = 0.006, Figure 4E). These results confirmed the decreased dopaminergic neurons in Map2k5^+/–^ mutant mice, consistent with the movement phenotypes displayed in behavior tests.

### Decreased Quantities of Map2k5 and TH Protein, but Not pErk5 in Map2k5^+/–^ Mutant Mice

**FIGURE 5:**
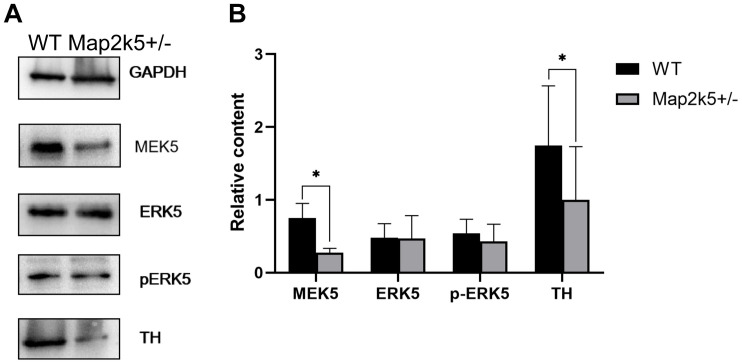
**(A)** Representative Western blots images of MAP2K5, ERK5, phosphorylated ERK5 (active), TH protein, and GAPDH are depicted. **(B)** Data from male WT and Map2k5^+/–^ mutant mice were pooled for this analysis. Bar graph is hemi-quantitative analysis of the Western blot, showing a significant decrease in MAP2K5 and TH (P_Map__2k__5_ = 0.023, P_TH_ = 0.013, respectively) in the Map2k5^+/–^ mutant mice. Results are plotted as mean with SEM (paired *t*-test, *n* = 4). Results are plotted as mean with SEM. Bars represent means with SEM. *P < 0.05.

To explore the mechanism of reduced dopaminergic neurons in Map2k5^+/–^ mutant mice, we measured the level of Map2k5–Erk5 pathway molecules and TH protein in ventral midbrain including dopaminergic neurons in the nigrostriatal pathway. Four female mice aged 21 weeks for each genotype were used. Proteins of MAP2K5 (MEK5), ERK5, phosphorylated ERK5 (active), TH, and GAPDH were stained (Figure 5A). The ratio of each protein was calculated by its relative quantity referred to the GAPDH band. The results implied a synchronized decrease in Map2k5 and TH proteins in the ventral midbrain (P_Map__2k__5_ = 0.023, P_TH_ = 0.013, respectively), while Erk5 was not reduced, and phosphorylated Erk5 was reduced without a statistical significance (*P* = 0.324, Figure 5B). It is possible that MAP2K5 deficiency decreases TH expression in an ERK5-independent manner. However, more studies are needed to clarify the molecular mechanism of this phenomenon.

### Dopamine Levels Were Not Altered in Striatum, but Its Substrates Were Accumulated

**FIGURE 6:**
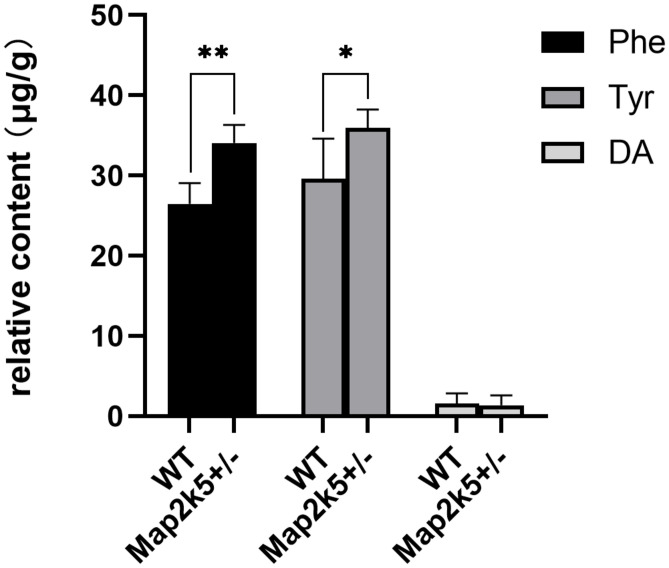
The values of neurochemicals in dopamine synthesis pathway are represented. Phe and Tyr are accumulated significantly in Map2k5^+/–^ mutant mice (*P* = 0.001, 0.033, respectively, *n* = 5). Results are plotted as mean with SEM. Bars represent means with SEM. *P < 0.05, **P < 0.01.

As TH is the key enzyme of dopamine synthesis and Map2k5-deficient mice showed dopaminergic neuron deficiency in the nigrostriatal pathway, we tried to investigate whether Map2k5 deficiency-induced TH reduction had an effect on neurotransmitters and their metabolites (Obara et al., 2016). We used HPLC to examine monoamine neurochemical systems in the striatum on five female 21-week-old mice per group. The substrates of DA in its synthesis pathway (Phe and Tyr) were accumulated significantly (*P* = 0.001, 0.033, respectively, Figure 6) in Map2k5^+/–^ female mutant mice compared with WT female mice, while the gross DA content decreased but without significant difference between WT and Map2k5^+/–^ mutant mice. Other neurotransmitters, such as Gln, ACh, serotonin, and its metabolite 5-HIAA were not altered in Map2k5^+/–^ mutant mice (Table 1). The accumulation of substrates of phenylalanine and tyrosine is consistent with the result of decreased TH enzyme in the nigrostriatal pathway. However, there was no statistical difference on the dopamine levels between Map2k5^+/–^ mutant and WT mice. This inconsistent finding may be due to two reasons. First, the dopamine level in the striatum was very low to be detected, beyond the linear dynamic range of the HPLC standard curve. This problem may be solved by other techniques like fluorometric detection (Ghani et al., 2021). Second, TH reduction in Map2k5-deficient mouse model was not strong enough to create a significant reduction in dopamine in the nigrostriatal pathway in Map2k5 mutant mice. Interestingly, previous studies showed that DA was not significantly reduced when TH protein level decreased significantly at 3 weeks in Th mutant mice. However, at 1 year old, when TH content reduced at 3% of WT mice, DA reduced to a significant level (*P* < 0.001) (Korner et al., 2015). Therefore, there may be a threshold of TH reduction when DA decreased into a significant level.

**TABLE 1:** Levels of phenylalanine (Phe), tyrosine (Tyr), dopamine (DA), epinephrine (Epi), serotonin, and its metabolite 5-HIAA in the striatum.

Neurochemicals	WT	Map2k5^+/–^	*P*-value
Phe	26.46 ± 1.17	34.04 ± 1.01	0.001**
Tyr	29.62 ± 2.24	35.95 ± 1.01	0.033*
DA	1.55 ± 0.59	1.34 ± 0.63	0.815
Epi	1.50 ± 0.62	1.07 ± 0.46	0.621
Gln	675.43 ± 189.03	574.01 ± 163.44	0.695
ACh	0.58 ± 0.17	0.22 ± 0.07	0.094
Serotonin	0.04 ± 0.00	0.03 ± 0.02	0.743
5-HIAA	0.35 ± 0.11	0.26 ± 0.05	0.478

The values of neurochemicals are represented in means ± SEM (μg/g) of the tissue (t-test, n = 5). *P < 0.05, **P < 0.01.

## Discussion

In this study, we described for the first time the movement phenotypes and underlying dopaminergic disturbance in a Map2k5-deficient mouse model. These behavioral changes were contrary to RLS/WED symptoms, but similar to PD in some aspects, including reduced circadian-dependent ambulatory locomotion, coordination, and balance ability. Similarly, reduced dopaminergic neurons and TH protein in the nigrostriatal pathway resembled some pathological aspects of PD. Actually, both diseases share many common features, such as high prevalence of comorbidity of RLS/WED with PD, dopaminergic dysregulation in the pathogenesis of both diseases, and good response to dopamine agonists (Splinter, 2007). This suggests similar mechanisms that might be shared by both disorders (Alonso-Navarro et al., 2019). In addition, SNPs of MAP2K5 have been shown to be associated with the reduced risk of RLS/WED (Winkelmann et al., 2007; Yang et al., 2011; Moore et al., 2014; Li et al., 2017), but increased the risk of tremor in PD (rs12593813, 61.0% vs. 46.5%, *p* = 0.001) (Gan-Or et al., 2015). This further indicates a close relationship, but probably opposite, effect of MAP2K5 in RLS/WED and PD. Also, PD patients usually present with odor problems, and previous studies reported deficits in odor discrimination in the conditional deletion of Erk5 mouse (Zou et al., 2012). Therefore, we hypothesized that the Map2k5–Erk5 pathway might be involved in the pathophysiology of both RLS/WED and PD.

Several *in vitro* studies found that the MAP2K5–ERK5 pathway was crucial in dopaminergic neuron survival. It protected dopaminergic neurons under both basal conditions and in response to oxidative stress (Cavanaugh et al., 2006). MAP2K5–ERK5 was also involved in Mn-induced cytotoxicity, altering neurons’ survival and DA production in MN9D cells (Ding et al., 2020). ERK5 was reported to induce ankrd1 for catecholamines biosynthesis and homeostasis in adrenal gland medullary cells (Obara et al., 2016). Thus, it is reasonable to hypothesize that reduced dopaminergic neurons and TH protein in our Map2k5-deficient mouse model might be due to decreased Map2k5–Erk5 pathway. However, p-Erk5 level remained unchanged in our Map2k5-deficient mouse. Based on the fact that the level of p-ERK5 dropped greatly after birth (Parmar et al., 2015) and Erk5 is the only present known substrate of Map2k5, the regulation of the Map2k5–Erk5 pathway on dopaminergic neuron probably occurred in the early developmental stage. Actually, Map2k5–Erk5 seems to be involved in protecting embryonic, but not mature, cortical neurons in an *in vitro* study (Liu et al., 2003, 2006). Also, conditional deletion of Erk5 in the nervous system was once reported for impaired neuron migration into the olfactory bulb during nervous system development at prenatal stages (Li et al., 2013). Since the heterozygous Map2k5 mutant mice already displayed reduction in TH^+^ dopaminergic cells significantly on postnatal 18 days in our study, it is plausible to think that the Map2k5–Erk5 pathway regulates the dopaminergic development in an earlier period and contributes to central nervous system disorders such as RLS/WED and PD. Actually, we aim to explore the Map2k5-deficient effect on dopaminergic neuron development of early neurogenesis in our future research.

Additionally, our results of behavioral tests displayed sexual distinctions. Here, we uncovered the nigrostriatal dopaminergic system disturbance in female Map2k5-deficient mice, while the underlying mechanism of stress-related alterations in sensorimotor response of male Map2k5-deficient mice, involving amygdala, hippocampus, and accumbens nuclei in the limbic system (Lalonde and Strazielle, 2017; Frau et al., 2019) is worth to be further studied.

There are limitations of our study. The dopamine level in the striatum was very low to be detected. As a matter of fact, several heterozygous samples’ DA concentrations were beyond the linear dynamic range of the HPLC standard curve. It could indeed account to difficulties to detect any difference in DA between groups. This problem may be solved by other techniques like fluorometric detection (Ghani et al., 2021). What is more, our results showed significant accumulation of substrates (Phe and Tyr) of dopamine before the step mediated by TH accompanied by insignificant reduction in DA, so we hypothesized that a threshold may exist where the reduction in TH induces DA decrease into a significant level.

## Conclusion

In conclusion, Map2k5-deficient mice manifest movement impairments, consistent with reductions in dopaminergic neurons and TH protein in the nigrostriatal dopaminergic system. These features confirmed that dopaminergic disturbance by the Map2k5–Erk5 pathway was involved in the pathogenesis of movement disorders like RLS/WED and PD. In addition, our Map2k5-deficient mouse model provides a unique tool to further investigate the role of the Map2k5–Erk5 pathway in other central nervous system disorders with dopamine deficits, such as schizophrenia.

## Data Availability Statement

The raw data supporting the conclusions of this article will be made available by the authors, without undue reservation.

## Ethics Statement

The animal study was reviewed and approved by the Ruijin Hospital, Shanghai Jiao Tong University School of Medicine.

## Author Contributions

YH contributed to the project configuration, experiment investigation, data analysis, and original manuscript description. JM contributed to the project supervision, data analysis manuscript modification, and funding acquisition. RM contributed to the manuscript modification and data curation. PW and QL participated in the experiment investigation, data analysis, and methodology support. All authors contributed to the article and approved the submitted version.

## Conflict of Interest

The authors declare that the research was conducted in the absence of any commercial or financial relationships that could be construed as a potential conflict of interest.

## Footnotes

1 http://crispor.tefor.net

2 https://www.mousephenotype.org/impress/index

## Abbreviations

ACh: acetylcholine
AMB: ambulatory activity
ASR: acoustic startle response
DA: dopamine
Epi: epinephrine
GAPDH: glyceraldehyde-3-phosphate dehydrogenase
Gln: glutamine
HCG: human chorionic gonadotropin
HPLC: high-performance liquid chromatography
KO: knockout
MAP2K5: mitogen-activated protein kinase kinase 5
P18d: postnatal 18 days
PBS: phosphate-buffered saline
PFA: paraformaldehyde
Phe: phenylalanine
PLMS: periodic limb movements
PPI: prepulse inhibition
qRT-PCR: semi-quantitative reverse transcriptase-PCR
RLS/WED: restless legs syndrome/Willis–Ekbom disease
SNc: substantia nigra pars compacta
SNP: single-nucleotide polymorphism
TH: tyrosine hydroxylase
TOT: total activity
Tyr: tyrosine
WT: wild type.
