# Supplementary material for: Map2k5-Deficient Mice Manifest Phenotypes and Pathological Changes of Dopamine Deficiency in the Central Nervous System
Source: Front Aging Neurosci. 2021 Jun 8;13:651638. doi: 10.3389/fnagi.2021.651638 (PMC8217467; doi:10.3389/fnagi.2021.651638)
Supplement: Supplementary file 1 [file Data_Sheet_1.ZIP › S2/T036804_Map2k5_off-target report.pdf]

Off Target Genotyping Report

Strain ID	T036804	Strain Type	KO(Cas9)	Genetic Background	C57BL/6JGpt
Designer	Tianjiao Wang	Gene Name	*Map2k5*		

1. Strategy of Genotyping

The potential off-target effect of several most susceptive loci were predicted on CRISPOR (http://crispor.tefor.net). According to the miss prediction of the website, the three sites with the highest miss probability were selected. Sanger sequencing was applied in all tests.

2. Primer Information

PCR No.	Primer No.	Primer Name	Sequence	Band Size
①S1-1 GC:33.4%	F1	CM004218.1-F1	AGGGCACACATTGTAGGTTTTCTG	Targeted:407bp
	R1	CM004218.1-R1	AATAGGCAGAGACTGCGGTTTCTC	
②S1-2	F2	CM004230.1-F2	CAACAGAACTTGCCCTTGGATTC	Targeted:668bp
	R2	CM004230.1-R2	TGGTGCTCAGCCCATTTCCTAAC	
③S1-3	F3	CM004216.1-F3	CCACTTGTGTTTGTATGTGTGCG	Targeted:459bp
	R3	CM004216.1-R3	TCTGCTTGCTCATGCACGAAACT	
④S3-1	F4	CM004219.1-F4	AATAAGCCTCTTCACCATCCTCC	Targeted:423bp
	R4	CM004219.1-R4	GCTTTCCCTCTAAAGGAATGAGG	
⑤S3-2	F5	CM004225.1-F5	ACTCATAGCAGCCATGAGTCTGC	Targeted:400bp
	R5	CM004225.1-R5	GAGAAGTTGAACTGGGACTGGACT	
⑥S3-3 GC:36.7%	F6	CM004233.1-F6	GGAAAATCCGCATACCACATG	Targeted:441bp
	R6	CM004233.1-R6	TGAGAGCTGTAATGTCTGTCCTGC	

Sequencing method

Primer Name	Sequence	Description
T014756-Map2k5-S1-tR1	AATAGGCAGAGACTGCGGTTTCTC	S1-1
T014756-Map2k5-S1-tF2	CAACAGAACTTGCCCTTGGATTC	S1-2
T014756-Map2k5-S1-tF3	CCACTTGTGTTTGTATGTGTGCG	S1-3
T014756-Map2k5-S3-tR1	GCTTTCCCTCTAAAGGAATGAGG	S3-1
T014756-Map2k5-S3-tF2	ACTCATAGCAGCCATGAGTCTGC	S3-2
T014756-Map2k5-S3-tF3	GGAAAATCCGCATACCACATG	S3-3

3. Gel Image & Conclusion

It was confirmed by PCR and sequencing (attached documents) that predicted off-target sites were wild-type sequences and no off-target was detected.

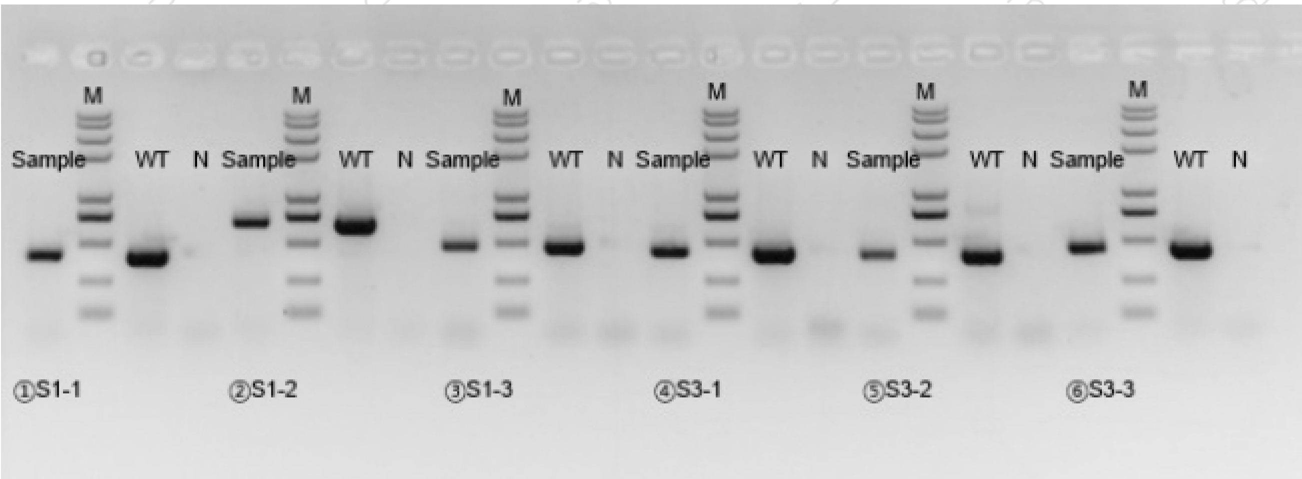

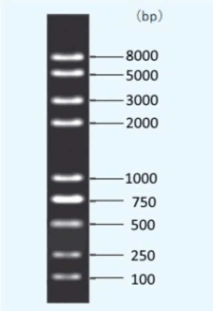

Note: WT: Wildype control; B: Blank control (ddH$_2$O); M: DNA Ladder

4. PCR Condition

PCR Reaction Component		
**Seg.**	**reaction component**	**Volume (μl)**
1	2 × Taq Master Mix， Dye Plus，(Vazyme P112-03)	12.5
2	ddH2O	9.5
3	Primer A(10pmol/μl)	1
4	Primer B(10pmol/μl)	1
5	Template(≈100ng/μl)	1
**PCR program**		

GemPharmatech Co.,Ltd

Seg.	Temp.	Time	Cycle
1	95℃	5min	
2	98℃	30s	20×
3	65℃（-0.5℃/cycle）	30s	
4	72℃	45s	
5	98℃	30s	20×
6	55℃	30s	
7	72℃	45s	
8	72℃	5min	
9	10℃	hold	

Reviewer:Ting Sun

Date:2021-4-2

GemPharmatech Co.,Ltd
