# Supplementary material for: Map2k5-Deficient Mice Manifest Phenotypes and Pathological Changes of Dopamine Deficiency in the Central Nervous System
Source: Front Aging Neurosci. 2021 Jun 8;13:651638. doi: 10.3389/fnagi.2021.651638 (PMC8217467; doi:10.3389/fnagi.2021.651638)
Supplement: Supplementary file 1 [file Data_Sheet_1.ZIP › supplementary material S3.pdf]

Supplementary material 3

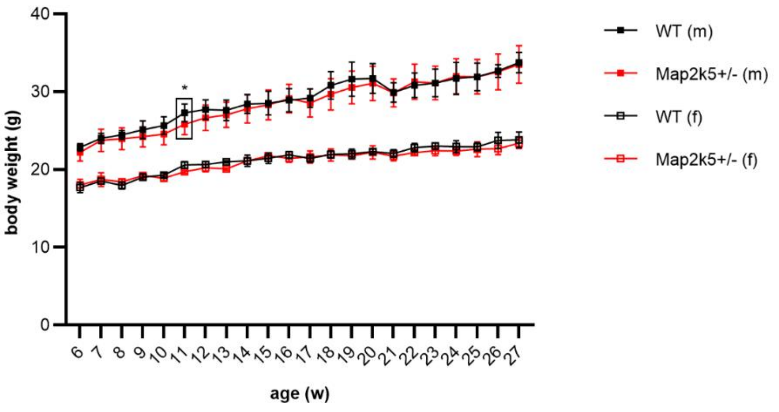

S3 Figure 1. Body weights were tracked from 6w to 27w. Statistical significance only in male Map2k5$^{+/-}$ mutant mice compared to WT littermates at 11 w (P=0.043, n=4). Results are plotted as mean with SEM. *P < 0.05.

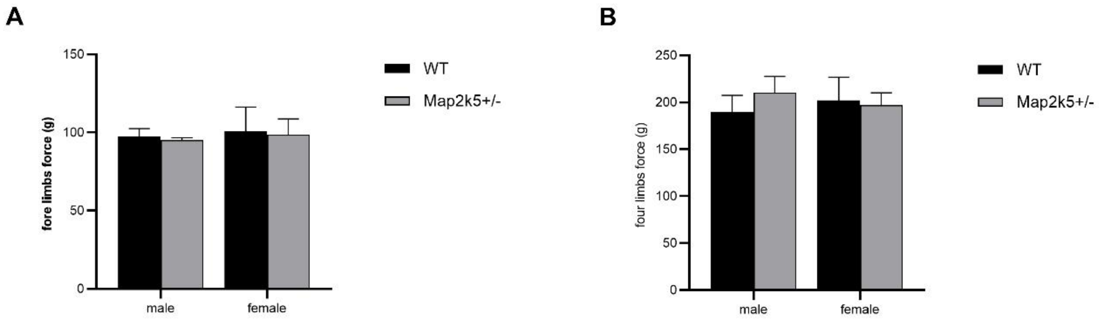

S3 Figure 2. A-B) Fore limbs' and four limbs' strength were recorded. No difference according to different genotypes was shown. Results are plotted as mean with SEM. Statistical significance (a=0.05, n=4). Bars represent means with SEM.
